# Tetra­phenyl­glycolide tetra­hydro­furan monosolvate

**DOI:** 10.1107/S2414314624012410

**Published:** 2025-01-07

**Authors:** Lubabalo Ndima, Eric Cyriel Hosten, Richard Betz

**Affiliations:** a Nelson Mandela University, Summerstrand Campus, Department of Chemistry, University Way, Summerstrand, PO Box 77000, Port Elizabeth, 6031, South Africa; Howard University, USA

**Keywords:** crystal structure, glycolide, C—H⋯O contacts

## Abstract

The title compound, C_28_H_20_O_4_·C_4_H_4_O, is the cyclic ester anhydride of benzilic acid. A disordered solvent mol­ecule is present in the structure. The asymmetric unit contains half the acid-derived mol­ecule. C—H⋯O contacts connect the constituents of the title compound into a three-dimensional network.

## Abstract 2

The title compound is the symmetric glycolide derived from benzilic acid featuring a disordered tetra­hydro­furan solvent mol­ecule in the crystal structure.

## Graphical abstract

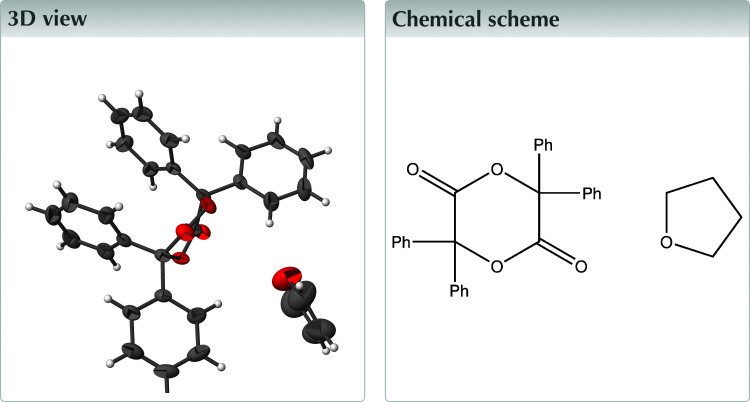

## Structure description

Heterocyclic compounds play a major role in biological systems, with sugars and the building blocks of DNA even being part of many high school curricula (Stryer, 1988 ▸). Owing to this finding, pharmaceutical research often employs aromatic and alicyclic compounds as leitmotifs from which potentially powerful new drugs can be derived. Against this backdrop it is not surprising that structural information about this class of mol­ecules, although already abundant, still constitutes a considerable focus of research up to this day. As part of our ongoing studies in this area (Nayak *et al.*, 2014 ▸; Mohamed *et al.*, 2023 ▸; Dayananda *et al.*, 2013 ▸; Lulama & Betz, 2015 ▸; Betz & Klüfers, 2007*a* ▸,*b* ▸,*c* ▸, 2008 ▸, 2009 ▸; Betz *et al.*, 2008 ▸, 2011 ▸, 2009 ▸, 2010 ▸; Potgieter *et al.*, 2011 ▸; Hosten & Betz, 2014 ▸; Averdunk *et al.*, 2021*a* ▸,*b* ▸), we sought to determine the crystal structure of the title compound that was obtained as a surprising outcome of an inorganic non-metal compound reaction. The crystal and mol­ecular structure of the solvent-free equivalent of the title compound are apparent in the literature (Shan *et al.*, 2005 ▸) as are other examples of symmetric cyclic ester anhydrides such as, *e.g.*, the ones derived from glycolic acid (Hutchison *et al.*, 2017 ▸; Belenkaya *et al.*, 1997 ▸), lactic acid (Chisholm *et al.*, 2000 ▸; van Hummel *et al.*, 1982 ▸; Belenkaya *et al.*, 1997 ▸) or 3-chloro­lactic acid (Kalelkar *et al.*, 2016 ▸), as well as examples of asymmetric members of this compound class such as the condensation products of lactic acid and mandelic acid (Nifant’ev *et al.*, 2020 ▸). The lactide of thiol­actic acid represents the only example where the mol­ecular and crystal structure of a thio­nated glycolide has been secured on grounds of diffraction studies on single crystals (Mangalum *et al.*, 2016 ▸).

**Figure 1:**
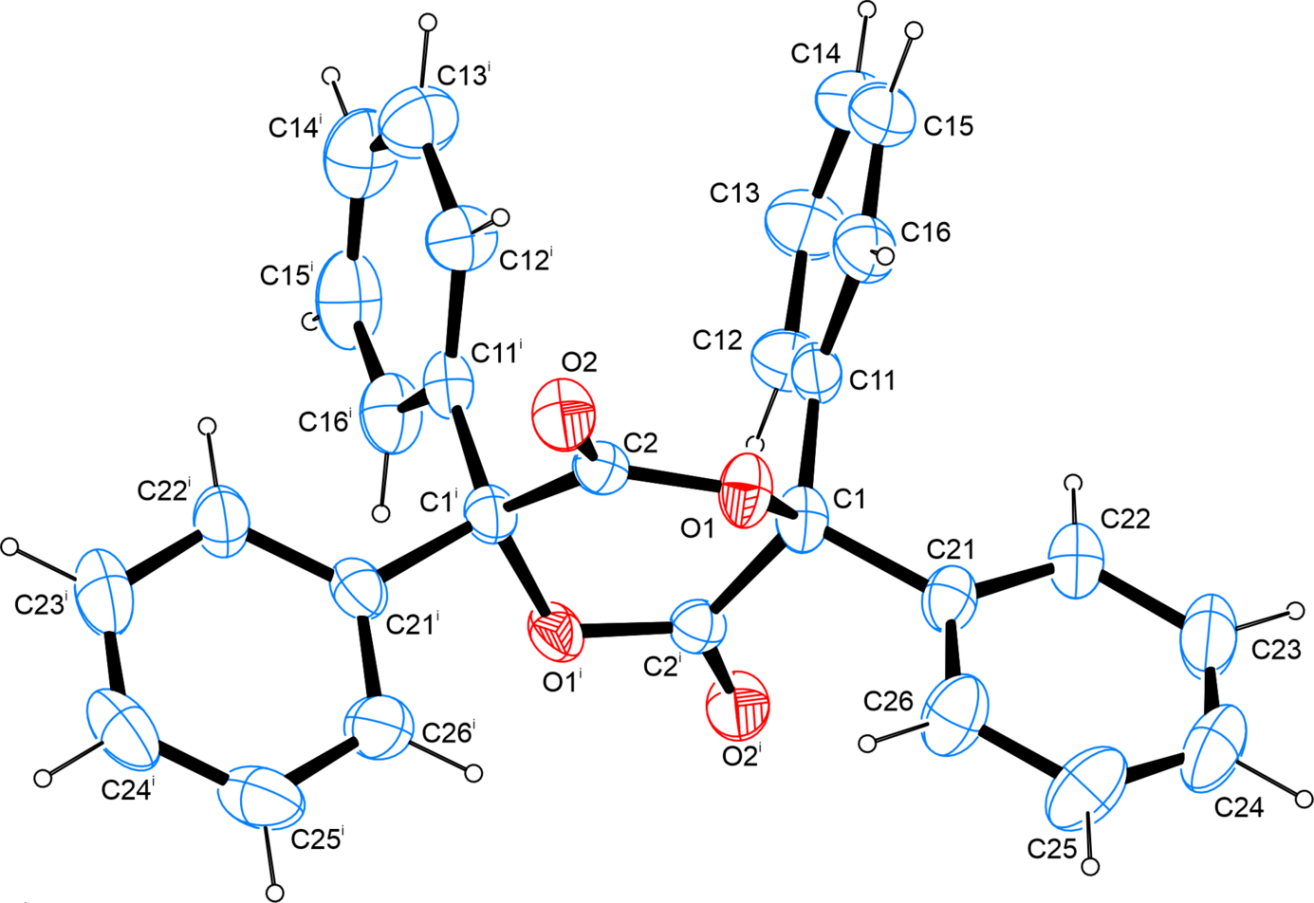
The mol­ecular structure of the title compound, with atom labels and anisotropic displacement ellipsoids (drawn at the 50% probability level). For clarity, the disordered THF mol­ecule has been omitted. Symmetry code: (i) *y*, *x*, −*z* + 1.

The title compound is the cyclic ester anhydride of benzilic acid. The structure refinement was conducted as a two-component inversion twin with a volume ratio of 75.1:24.9. The asymmetric unit contains half a mol­ecule. One disordered mol­ecule of tetra­hydro­furan is also present in the crystal structure. The C—O and C=O bond lengths are found at 1.467 (3) and 1.340 (3) Å, respectively, and, therefore, are in good agreement with values reported for other cyclic lactides whose mol­ecular and crystal structures have been determined on grounds of diffraction studies on single crystals and whose metrical parameters have been deposited with the Cambridge Structural Database (Groom *et al.*, 2016 ▸). A conformational analysis of the six-membered heterocycle according to Cremer & Pople (1975 ▸) shows the latter to adopt a confirmation almost exactly in between a ^4^*T*_2_ (^O1i^*T*_C1_) as well as a *B*_C1,C1i_ conformation (Boeyens, 1978 ▸). The phenyl rings are orientated almost perpendicular to one another as the least-squares planes, as defined by the respective carbon atoms of the aromatic moieties, enclose an angle of 85.34 (16)° (Fig. 1 ▸).

**Figure 2:**
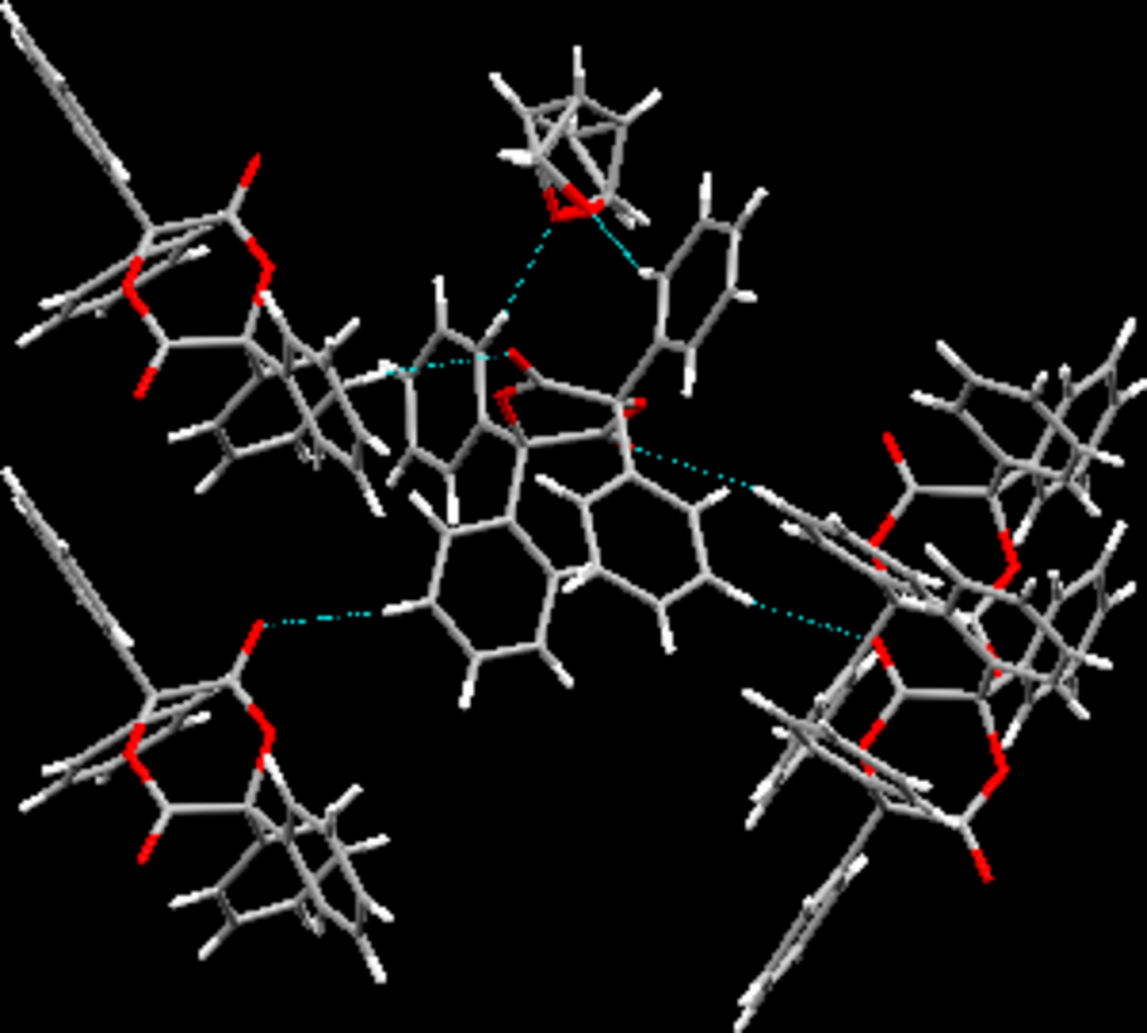
Inter­molecular contacts, viewed approximately along [110].

In the crystal, there are C—H⋯O contacts (Table 1 ▸) whose range falls by more than 0.1 Å below the sum of the van der Waals radii of the atoms participating in them. These are supported by one hydrogen atom each in the *ortho*-position on two of the aromatic systems as donors and, invariably, the oxygen atom of the solvent mol­ecule as acceptor. A second type of C—H⋯O contact is found between one hydrogen atom each in *meta*-position on the remaining two phenyl groups (that had not participated in the previously described contacts) as donors and the two carbonylic oxygen atoms as acceptors. In terms of graph-set analysis (Etter *et al.*, 1990 ▸; Bernstein *et al.*, 1995 ▸), the descriptor for these C—H⋯O contacts requires a *DDC^1^_1_(7) 
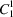
(7)* descriptor on the unary level. In total, these inter­actions connect the constituents present in the crystal structure of the title compound to a three-dimensional network. Furthermore, one C—H⋯π contact is apparent between one of the hydrogen atoms in the *meta*-position on one of the phenyl groups giving rise to the C—H⋯O inter­actions towards the solvent mol­ecule as donor and one of the aromatic systems of an aromatic system that gives rise to the contacts involving the carbonyl group. In addition, the structure is further consolidated by π-stacking inter­actions with the shortest distance between two centres of gravity measured at 3.8915 (19) Å in between two phenyl groups, giving rise to the C—H⋯O contacts towards the solvent mol­ecule present in the crystal structure (Fig. 2 ▸).

## Synthesis and crystallization

The compound was obtained by reacting penta­carbonyl­rhenium(I) chloride and the hydrido­spiro­phospho­rane derived from benzilic acid in the mixed solvents of THF/benzene/di­ethyl­ether. Crystals suitable for the diffraction study were obtained upon concentrating the reaction mixture and subsequent storage at room temperature.

## Refinement

Crystal data, data collection and structure refinement details are summarized in Table 2 ▸. The modelling of the disordered THF mol­ecule was conducted applying RIGU and ISOR instructions.

## Supplementary Material

Crystal structure: contains datablock(s) I. DOI: 10.1107/S2414314624012410/bv4054sup1.cif

Structure factors: contains datablock(s) I. DOI: 10.1107/S2414314624012410/bv4054Isup2.hkl

Supporting information file. DOI: 10.1107/S2414314624012410/bv4054Isup3.cml

CCDC reference: 2412616

Additional supporting information:  crystallographic information; 3D view; checkCIF report

## Figures and Tables

**Table 1:** Hydrogen-bond geometry (Å, °) *Cg*1 is the centroid of carbon atoms C21–C26.

*D*—H⋯*A*	*D*—H	H⋯*A*	*D*⋯*A*	*D*—H⋯*A*
C12—H12⋯O2^i^	0.95	2.55	3.185 (4)	124
C15—H15⋯O2^ii^	0.95	2.57	3.494 (4)	166
C26—H26⋯O3	0.95	2.17	3.060 (7)	155
C13—H13⋯*Cg*1^iii^	0.95	2.90	3.799 (4)	158

Symmetry codes: (i) 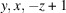; (ii) 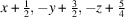; (iii) 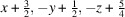.

**Table 2:** Experimental details

Crystal data	
Chemical formula	C_28_H_20_O_4_·C_4_H_4_O
*M*_r_	488.51
Crystal system, space group	Tetragonal, *P*4_3_2_1_2
Temperature (K)	200
*a*, *c* (Å)	9.5725 (3), 27.5760 (11)
*V* (Å^3^)	2526.86 (18)
*Z*	4
Radiation type	Mo *K*α
μ (mm^−1^)	0.09
Crystal size (mm)	0.24 × 0.23 × 0.15

Data collection	
Diffractometer	Bruker D8 Quest CCD
Absorption correction	Multi-scan (*SADABS*; Krause *et al.*, 2015 ▸)
*T*_min_, *T*_max_	0.717, 0.746
No. of measured, independent and observed [*I* > 2σ(*I*)] reflections	62563, 2796, 2460
*R*_int_	0.080
(sin θ/λ)_max_ (Å^−1^)	0.641

Refinement	
*R*[*F*^2^ > 2σ(*F*^2^)], *wR*(*F*^2^), *S*	0.056, 0.176, 1.09
No. of reflections	2796
No. of parameters	182
No. of restraints	60
H-atom treatment	H-atom parameters constrained
Δρ_max_, Δρ_min_ (e Å^−3^)	0.61, −0.49
Absolute structure	Refined as an inversion twin

Computer programs: *APEX2* and *SAINT* (Bruker, 2014 ▸), *SHELXS97* (Sheldrick 2008 ▸), *SHELXL2019/3* (Sheldrick, 2015 ▸), *ORTEP-3 for Windows* (Farrugia, 2012 ▸), *Mercury* (Macrae *et al.*, 2020 ▸) and *PLATON* (Spek, 2020 ▸).

## full crystallographic data

### 3,3,6,6-Tetraphenyl-1,4-dioxane-2,5-dione tetrahydrofuran monosolvate Crystal data

**Table:**

C_28_H_20_O_4_·C_4_H_4_O	*D*_x_ = 1.284 Mg m^−^^3^
*M**_r_* = 488.51	Mo *K*α radiation, λ = 0.71073 Å
Tetragonal, *P*4_3_2_1_2	Cell parameters from 9736 reflections
*a* = 9.5725 (3) Å	θ = 2.3–27.2°
*c* = 27.5760 (11) Å	µ = 0.09 mm^−^^1^
*V* = 2526.86 (18) Å^3^	*T* = 200 K
*Z* = 4	Block, colourless
*F*(000) = 1024	0.24 × 0.23 × 0.15 mm

### 3,3,6,6-Tetraphenyl-1,4-dioxane-2,5-dione tetrahydrofuran monosolvate Data collection

**Table:**

Bruker D8 Quest CCD diffractometer	2460 reflections with *I* > 2σ(*I*)
φ and ω scans	*R*_int_ = 0.080
Absorption correction: multi-scan (SADABS; Krause *et al.*, 2015)	θ_max_ = 27.1°, θ_min_ = 2.3°
*T*_min_ = 0.717, *T*_max_ = 0.746	*h* = −12→11
62563 measured reflections	*k* = −12→11
2796 independent reflections	*l* = −34→35

### 3,3,6,6-Tetraphenyl-1,4-dioxane-2,5-dione tetrahydrofuran monosolvate Refinement

**Table:**

Refinement on *F*^2^	Secondary atom site location: difference Fourier map
Least-squares matrix: full	Hydrogen site location: inferred from neighbouring sites
*R*[*F*^2^ > 2σ(*F*^2^)] = 0.056	H-atom parameters constrained
*wR*(*F*^2^) = 0.176	*w* = 1/[σ^2^(*F*_o_^2^) + (0.1205*P*)^2^ + 0.5364*P*] where *P* = (*F*_o_^2^ + 2*F*_c_^2^)/3
*S* = 1.09	(Δ/σ)_max_ < 0.001
2796 reflections	Δρ_max_ = 0.61 e Å^−^^3^
182 parameters	Δρ_min_ = −0.49 e Å^−^^3^
60 restraints	Absolute structure: Refined as an inversion twin
Primary atom site location: structure-invariant direct methods	Absolute structure parameter: 0 (2)

### 3,3,6,6-Tetraphenyl-1,4-dioxane-2,5-dione tetrahydrofuran monosolvate Special details

**Table:**

Geometry. All esds (except the esd in the dihedral angle between two l.s. planes) are estimated using the full covariance matrix. The cell esds are taken into account individually in the estimation of esds in distances, angles and torsion angles; correlations between esds in cell parameters are only used when they are defined by crystal symmetry. An approximate (isotropic) treatment of cell esds is used for estimating esds involving l.s. planes.
Refinement. Refined as a 2-component inversion twin. The carbon-bound H atoms were placed in calculated positions (C–H 0.95 Å for aromatic carbon atoms and methylene groups) and were included in the refinement in the riding model approximation, with *U*(H) set to 1.2*U*_eq_(C).

### 3,3,6,6-Tetraphenyl-1,4-dioxane-2,5-dione tetrahydrofuran monosolvate Fractional atomic coordinates and isotropic or equivalent isotropic displacement parameters (Å^2^)

**Table:**

	*x*	*y*	*z*	*U*_iso_*/*U*_eq_	Occ. (<1)
O1	0.4588 (2)	0.4898 (2)	0.54864 (6)	0.0319 (5)	
O2	0.2991 (2)	0.6508 (2)	0.53907 (7)	0.0368 (5)	
C1	0.5915 (3)	0.4283 (3)	0.53272 (9)	0.0283 (6)	
C2	0.3893 (3)	0.5818 (3)	0.52110 (9)	0.0260 (5)	
C11	0.7086 (3)	0.5314 (3)	0.54213 (9)	0.0298 (6)	
C12	0.8216 (3)	0.5456 (3)	0.51104 (11)	0.0379 (7)	
H12	0.825856	0.490886	0.482324	0.046*	
C13	0.9293 (4)	0.6394 (4)	0.52150 (13)	0.0492 (8)	
H13	1.006380	0.648771	0.500032	0.059*	
C14	0.9226 (4)	0.7188 (4)	0.56365 (14)	0.0538 (9)	
H14	0.995517	0.782443	0.571219	0.065*	
C15	0.8095 (4)	0.7051 (4)	0.59457 (12)	0.0505 (9)	
H15	0.804601	0.760230	0.623176	0.061*	
C16	0.7043 (4)	0.6123 (3)	0.58411 (11)	0.0401 (7)	
H16	0.627644	0.603108	0.605757	0.048*	
C21	0.6034 (3)	0.2930 (3)	0.56191 (9)	0.0310 (6)	
C22	0.7258 (3)	0.2549 (3)	0.58459 (10)	0.0392 (7)	
H22	0.805731	0.313339	0.582405	0.047*	
C23	0.7323 (4)	0.1301 (4)	0.61081 (12)	0.0503 (9)	
H23	0.816855	0.103912	0.626369	0.060*	
C24	0.6182 (4)	0.0460 (4)	0.61418 (12)	0.0511 (9)	
H24	0.623033	−0.038158	0.632355	0.061*	
C25	0.4949 (4)	0.0829 (4)	0.59113 (14)	0.0516 (9)	
H25	0.415367	0.023889	0.593483	0.062*	
C26	0.4876 (4)	0.2054 (4)	0.56472 (13)	0.0444 (8)	
H26	0.403565	0.229794	0.548486	0.053*	
O3	0.2067 (8)	0.1892 (10)	0.5120 (3)	0.095 (3)	0.5
C31	0.2086 (7)	0.0816 (11)	0.4766 (3)	0.127 (6)	0.5
H31	0.289498	0.042872	0.461821	0.153*	0.5
C32	0.0683 (8)	0.0420 (8)	0.4671 (3)	0.091 (3)	0.5
H32	0.038849	−0.027875	0.444817	0.109*	0.5
C33	−0.0203 (6)	0.1251 (9)	0.4966 (3)	0.082 (3)	0.5
H33	−0.119378	0.120617	0.497626	0.099*	0.5
C34	0.0653 (9)	0.2161 (8)	0.5244 (3)	0.097 (4)	0.5
H34	0.033481	0.283137	0.547268	0.116*	0.5

### 3,3,6,6-Tetraphenyl-1,4-dioxane-2,5-dione tetrahydrofuran monosolvate Atomic displacement parameters (Å^2^)

**Table:**

	*U*^11^	*U*^22^	*U*^33^	*U*^12^	*U*^13^	*U*^23^
O1	0.0334 (10)	0.0385 (11)	0.0238 (8)	0.0097 (8)	0.0075 (7)	0.0069 (8)
O2	0.0376 (11)	0.0416 (11)	0.0311 (10)	0.0111 (9)	0.0033 (8)	−0.0019 (9)
C1	0.0287 (13)	0.0338 (13)	0.0223 (12)	0.0089 (11)	0.0041 (10)	0.0025 (10)
C2	0.0259 (12)	0.0266 (12)	0.0255 (12)	−0.0022 (10)	0.0001 (9)	−0.0012 (9)
C11	0.0360 (14)	0.0294 (12)	0.0241 (12)	0.0061 (11)	−0.0051 (11)	0.0009 (10)
C12	0.0380 (16)	0.0394 (16)	0.0364 (15)	−0.0027 (12)	0.0019 (12)	−0.0064 (12)
C13	0.0434 (18)	0.051 (2)	0.0529 (19)	−0.0079 (16)	−0.0027 (15)	−0.0047 (15)
C14	0.061 (2)	0.0406 (17)	0.060 (2)	−0.0069 (17)	−0.0195 (18)	−0.0056 (16)
C15	0.073 (3)	0.0374 (16)	0.0415 (16)	0.0089 (16)	−0.0189 (16)	−0.0097 (14)
C16	0.0496 (18)	0.0400 (16)	0.0306 (14)	0.0108 (14)	−0.0041 (13)	−0.0054 (12)
C21	0.0364 (15)	0.0320 (13)	0.0247 (12)	0.0053 (11)	0.0049 (10)	0.0039 (10)
C22	0.0412 (16)	0.0417 (16)	0.0349 (14)	0.0062 (14)	−0.0043 (12)	0.0082 (12)
C23	0.063 (2)	0.0485 (19)	0.0395 (16)	0.0148 (18)	−0.0063 (16)	0.0110 (14)
C24	0.073 (3)	0.0398 (17)	0.0403 (16)	0.0146 (17)	0.0148 (17)	0.0148 (14)
C25	0.051 (2)	0.0387 (17)	0.065 (2)	0.0018 (16)	0.0213 (17)	0.0127 (16)
C26	0.0357 (16)	0.0423 (17)	0.0551 (19)	0.0058 (13)	0.0056 (14)	0.0129 (15)
O3	0.059 (3)	0.113 (5)	0.113 (6)	0.001 (3)	−0.009 (4)	−0.019 (4)
C31	0.110 (7)	0.118 (8)	0.154 (8)	0.011 (5)	0.026 (6)	−0.027 (6)
C32	0.090 (5)	0.091 (5)	0.092 (5)	0.014 (4)	−0.010 (4)	−0.028 (4)
C33	0.070 (4)	0.102 (5)	0.074 (5)	0.021 (4)	−0.008 (4)	−0.015 (4)
C34	0.066 (5)	0.092 (6)	0.132 (7)	0.001 (4)	0.017 (5)	−0.024 (5)

### 3,3,6,6-Tetraphenyl-1,4-dioxane-2,5-dione tetrahydrofuran monosolvate Geometric parameters (Å, º)

**Table:**

O1—C2	1.340 (3)	C22—C23	1.398 (4)
O1—C1	1.467 (3)	C22—H22	0.9500
O2—C2	1.195 (3)	C23—C24	1.360 (6)
C1—C11	1.516 (4)	C23—H23	0.9500
C1—C21	1.529 (4)	C24—C25	1.386 (6)
C1—C2^i^	1.533 (3)	C24—H24	0.9500
C11—C12	1.387 (4)	C25—C26	1.382 (5)
C11—C16	1.393 (4)	C25—H25	0.9500
C12—C13	1.397 (5)	C26—H26	0.9500
C12—H12	0.9500	O3—C31	1.4200
C13—C14	1.391 (5)	O3—C34	1.4200
C13—H13	0.9500	C31—C32	1.4200
C14—C15	1.384 (6)	C31—H31	0.9500
C14—H14	0.9500	C32—C33	1.4200
C15—C16	1.374 (5)	C32—H32	0.9500
C15—H15	0.9500	C33—C34	1.4200
C16—H16	0.9500	C33—H33	0.9500
C21—C22	1.377 (4)	C34—H34	0.9500
C21—C26	1.392 (5)		
			
C2—O1—C1	121.62 (19)	C26—C21—C1	118.7 (3)
O1—C1—C11	109.1 (2)	C21—C22—C23	119.9 (3)
O1—C1—C21	104.3 (2)	C21—C22—H22	120.0
C11—C1—C21	114.0 (2)	C23—C22—H22	120.0
O1—C1—C2^i^	109.6 (2)	C24—C23—C22	120.4 (3)
C11—C1—C2^i^	111.6 (2)	C24—C23—H23	119.8
C21—C1—C2^i^	107.9 (2)	C22—C23—H23	119.8
O2—C2—O1	119.2 (2)	C23—C24—C25	120.1 (3)
O2—C2—C1^i^	122.9 (2)	C23—C24—H24	119.9
O1—C2—C1^i^	117.9 (2)	C25—C24—H24	119.9
C12—C11—C16	118.9 (3)	C26—C25—C24	120.0 (4)
C12—C11—C1	122.3 (2)	C26—C25—H25	120.0
C16—C11—C1	118.8 (3)	C24—C25—H25	120.0
C11—C12—C13	120.7 (3)	C25—C26—C21	120.0 (3)
C11—C12—H12	119.6	C25—C26—H26	120.0
C13—C12—H12	119.6	C21—C26—H26	120.0
C14—C13—C12	119.3 (3)	C31—O3—C34	108.0
C14—C13—H13	120.3	O3—C31—C32	108.0
C12—C13—H13	120.3	O3—C31—H31	126.0
C15—C14—C13	120.0 (3)	C32—C31—H31	126.0
C15—C14—H14	120.0	C31—C32—C33	108.0
C13—C14—H14	120.0	C31—C32—H32	126.0
C16—C15—C14	120.3 (3)	C33—C32—H32	126.0
C16—C15—H15	119.8	C34—C33—C32	108.0
C14—C15—H15	119.8	C34—C33—H33	126.0
C15—C16—C11	120.8 (3)	C32—C33—H33	126.0
C15—C16—H16	119.6	C33—C34—O3	108.0
C11—C16—H16	119.6	C33—C34—H34	126.0
C22—C21—C26	119.5 (3)	O3—C34—H34	126.0
C22—C21—C1	121.8 (3)		
			
C2—O1—C1—C11	80.3 (3)	O1—C1—C21—C22	−134.4 (3)
C2—O1—C1—C21	−157.5 (2)	C11—C1—C21—C22	−15.5 (4)
C2—O1—C1—C2^i^	−42.2 (3)	C2^i^—C1—C21—C22	109.1 (3)
C1—O1—C2—O2	−164.4 (3)	O1—C1—C21—C26	46.7 (3)
C1—O1—C2—C1^i^	16.7 (3)	C11—C1—C21—C26	165.6 (3)
O1—C1—C11—C12	−144.0 (3)	C2^i^—C1—C21—C26	−69.8 (3)
C21—C1—C11—C12	99.9 (3)	C26—C21—C22—C23	−1.0 (5)
C2^i^—C1—C11—C12	−22.7 (4)	C1—C21—C22—C23	−179.8 (3)
O1—C1—C11—C16	37.7 (3)	C21—C22—C23—C24	−0.1 (5)
C21—C1—C11—C16	−78.5 (3)	C22—C23—C24—C25	0.7 (5)
C2^i^—C1—C11—C16	158.9 (2)	C23—C24—C25—C26	−0.1 (5)
C16—C11—C12—C13	−0.1 (5)	C24—C25—C26—C21	−1.1 (5)
C1—C11—C12—C13	−178.4 (3)	C22—C21—C26—C25	1.6 (5)
C11—C12—C13—C14	0.1 (5)	C1—C21—C26—C25	−179.5 (3)
C12—C13—C14—C15	−0.4 (5)	C34—O3—C31—C32	0.0
C13—C14—C15—C16	0.7 (5)	O3—C31—C32—C33	0.0
C14—C15—C16—C11	−0.6 (5)	C31—C32—C33—C34	0.0
C12—C11—C16—C15	0.3 (4)	C32—C33—C34—O3	0.0
C1—C11—C16—C15	178.7 (3)	C31—O3—C34—C33	0.0

Symmetry code: (i) *y*, *x*, −*z*+1.

### 3,3,6,6-Tetraphenyl-1,4-dioxane-2,5-dione tetrahydrofuran monosolvate Hydrogen-bond geometry (Å, º)

*Cg*1 is the centroid of carbon atoms C21–C26.

**Table:**

*D*—H···*A*	*D*—H	H···*A*	*D*···*A*	*D*—H···*A*
C12—H12···O2^i^	0.95	2.55	3.185 (4)	124
C15—H15···O2^ii^	0.95	2.57	3.494 (4)	166
C26—H26···O3	0.95	2.17	3.060 (7)	155
C13—H13···*Cg*1^iii^	0.95	2.90	3.799 (4)	158

Symmetry codes: (i) *y*, *x*, −*z*+1; (ii) *x*+1/2, −*y*+3/2, −*z*+5/4; (iii) *x*+3/2, −*y*+1/2, −*z*+5/4.
